# Cardioprotective Roles of Endothelial Progenitor Cell-Derived Exosomes

**DOI:** 10.3389/fcvm.2021.717536

**Published:** 2021-08-26

**Authors:** Cai-Yu Zeng, Jia Xu, Xin Liu, Yuan-Qiang Lu

**Affiliations:** ^1^Department of Emergency Medicine, The First Affiliated Hospital, School of Medicine, Zhejiang University, Hangzhou, China; ^2^Department of Geriatrics, The First Affiliated Hospital, School of Medicine, Zhejiang University, Hangzhou, China; ^3^Zhejiang Provincial Key Laboratory for Diagnosis and Treatment of Aging and Physic-Chemical Injury Diseases, The First Affiliated Hospital, School of Medicine, Zhejiang University, Hangzhou, China

**Keywords:** cardiovascular diseases, EPC-derived exosomes, anti-inflammatory, anti-apoptosis, cardiac fibrosis, cell regeneration

## Abstract

With the globally increasing prevalence, cardiovascular diseases (CVDs) have become the leading cause of mortality. The transplantation of endothelial progenitor cells (EPCs) holds a great promise due to their potential for vasculogenesis, angiogenesis, and protective cytokine release, whose mechanisms are essential for CVD therapies. In reality, many investigations have attributed the therapeutic effects of EPC transplantation to the secretion of paracrine factors rather than the differentiation function. Of note, previous studies have suggested that EPCs could also release exosomes (diameter range of 30–150 nm), which carry various lipids and proteins and are abundant in microRNAs. The EPC-derived exosomes (EPC-EXs) were reported to act on the heart and blood vessels and were implicated in anti-inflammation, anti-oxidation, anti-apoptosis, the inhibition of endothelial-to-mesenchymal transition (EndMT), and cardiac fibrosis, as well as anti-vascular remodeling and angiogenesis, which were considered as protective effects against CVDs. In this review, we summarize the current knowledge on using EPC-EXs as therapeutic agents and provide a detailed description of their identified mechanisms of action to promote the prognosis of CVDs.

## Graphical Abstract

EPC-EXs: The hallmarks and pleiotropic functions. EPC-EXs are extracellular vesicles generated by EPCs and they carry lipids, proteins, nucleic acids, metabolites, and some EPC markers. They mediate intercellular communication and affect various aspects of cell biology. 
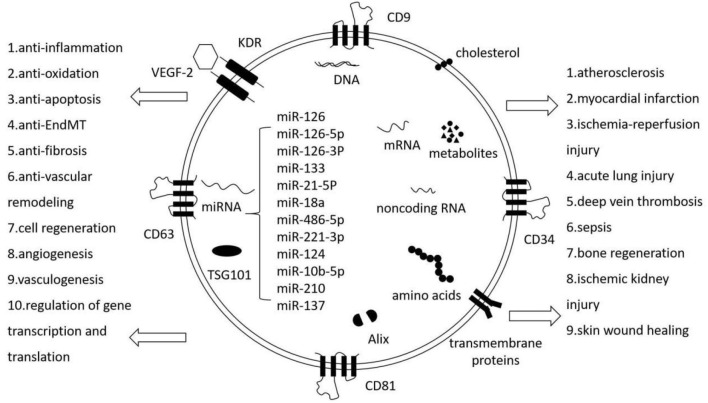

## Introduction

Cardiovascular diseases (CVDs), especially acute myocardial infarction (AMI), are a primary cause of death worldwide, responsible for an estimated 31% of all deaths, and 17.9 million lives each year. Events that contribute to AMI and subsequent complications are multifaceted, such as thrombosis, the activation of renin–angiotensin–aldosterone system (RAAS), cytokine release, oxidative stress, inflammatory effects, endothelial dysfunction, and ventricular remodeling (1–3). Endothelial dysfunction is an early marker of CVDs and the following complications. Many factors such as endothelial nitric oxide synthase (eNOS) uncoupling and inflammatory cytokines reduce endothelial FGFR1 and endothelial glucocorticoid receptor expression and activate transforming growth factor-beta (TGF-β) and Wnt signaling. TGF-β activates Smad proteins and shuttles to the nucleus to interact with Snail, Twist, and Slug to induce endothelial-to-mesenchymal transition (EndMT) (4, 5). Wnt signaling contributes to the disruption of cytokine and chemokine homeostasis, and results in EndMT (6). Mounting evidence indicates that EndMT is involved in CVDs, including pulmonary hypertension, atherosclerosis, and valvular disease. Reperfusion strategies, especially bypass surgery and percutaneous coronary intervention (PCI), are currently the main treatments for AMI, and are accountable for a significant reduction of AMI-related morbidity and mortality. With the extensive development of cell therapies for clinical application, stem cell-based AMI therapy has shown a great promise clinically in regenerating damaged myocardium and enhancing cardiac function in animal models and patients (7, 8). Endothelial progenitor cells (EPCs) are considered as highly potent regenerating cells with strong proliferative ability in response to tissue ischemia or hypoxia. For example, intravenously injected EPCs could incorporate into post-infarct myocardium, differentiate into new blood vessel in the infarct bed (vasculogenesis), and trigger the proliferation of pre-existing vasculature (angiogenesis) (9, 10). In fact, the general consensus is that only a minority of EPCs survive and engraft after transplantation because of potential immunological rejection, chromosomal variation, embolus formation, and so on (11). Nonetheless, EPC transplantation was shown to improve function and prognosis in AMI to an extent, and this was attributed to EPCs secreting paracrine factors and the more recently studied exosomes (12, 13). EPC-derived exosomes (EPC-EXs) were critical paracrine factors in promoting endothelial dysfunction, such as anti-inflammation, anti-oxidation, anti-apoptosis, inhibition of EndMT and cardiac fibrosis, anti-vascular remodeling, and angiogenesis in CVDs. EPC-EXs also showed protective effects in acute pulmonary injury, sepsis, ischemic kidney injury, skin wound healing, and so on. Except for exosomes marker, EPC-EXs also expressed many molecules characteristic of EPCs signatures such as CD34, vascular endothelial growth factor receptor (VEGFR)-2, and kinase insert domain receptor (KDR), and presented functions of EPCs such as enhancing EPCs proliferation, migration, and angiogenic tubule formation as well (**Graphical Abstract**). In this review, we summarize our current knowledge on using EPC-EXs as therapeutic agents for CVDs and discuss the identified mechanisms through which they exert their effects.

## EPC-Derived Exosomes

### Characteristics of Exosomes

Extracellular vesicles (EVs), which have a key role in intercellular and even interorganismal communication, include three subpopulations based on intracellular origin and size: microvesicles, exosomes, and apoptosomes. Microvesicles (diameter range of 50 nm−1 μm, and in some cases up to 10 μm) originate from the fission of the plasma membrane and direct outwards budding. Apoptosomes (diameter range of 500 nm−5 μm) resulted from the apoptotic cell disassembly. Compared with other secreted vesicles, exosomes have much better-defined roles in several biological and pathological processes. Exosomes (diameter range of 30–150 nm) carry various lipids and proteins and are abundant in mRNA, DNA, microRNA, long non-coding RNA, and further nucleic acid species (14). In general, exosome biogenesis consists of three steps: the formation of endocytic vesicles from the plasma membrane, the inward budding of the endosomes resulting in intracellular multivesicular bodies (MVBs) that consist of intraluminal vesicles (ILVs), and the release of these MVBs known as exosomes (15). Exosomes have been isolated from breast milk, blood, urine, cerebrospinal fluid, saliva, etc. by differential centrifugation, monoclonal antibody-based methods, and ultrafiltration. Also, the previous research has demonstrated a workflow to quantitatively analyze proteins of extracellular vesicle subgroups by an optimized method combining polymer-based precipitation and size exclusion chromatography (16). It was reported that various cells release exosomes, including mesenchymal stem cells (MSCs), neurons, T lymphocytes, B lymphocytes, endothelial cells (ECs), EPCs, tumor cells, and others. Since this discovery, numerous investigations have identified exosomes as a means of intercellular communication with a beneficial role in physiological processes, such as immune response, inflammation, and cell regeneration (17–19). They were also implicated in the pathogenesis of atherosclerosis, vascular remodeling, and thrombosis (20), as well as the development and deterioration of diseases such as tumors, Alzheimer's disease, and HIV-1 infection (21–23). Exosomes also contained organ-protective antifibrotic microRNAs, such as miR-29 and let-7s. A previous study has demonstrated that exosome-encapsulated miR-29 attenuated kidney fibrosis by downregulation of YY1 and TGF-β3. Transcription factor YY1 directly upregulated αSMA and collagen. TGF-β3 activated SMAD-based or non-SMAD-based pathways, resulting in fibrosis (24–26). Other non-coding RNAs included circular RNA (circRNiAs), P-element induced Wimpy testis (PIWI)-interacting RNAs (piRNAs), and long non-coding RNA (lncRNAs) except miRNAs. CircRNAs were a class of endogenous non-coding RNAs that formed a closed continuous loop without 5′ caps and 3′ poly tails. CircRNAs were found to regulate the transcription and function of miRNA-target genes and participated in the pathogenesis of multiple CVDs (27). piRNAs were a class of small RNAs that were 24–31 nucleotides in length. piRNAs silenced gene expression by interacting with PIWI proteins and guiding them to silence transposable elements (28). LncRNAs were non-protein-coding RNAs longer than 200 nucleotides. LncRNAs regulated gene expression at transcriptional, post-transcriptional, and epigenetic levels (29). IV-injected exosomes were detected in the spleen, followed by the liver, then the lungs and kidneys, but the brain, heart, and muscle showed lower amounts than others. Also, the curve of exosomes distribution presented a rapid distribution phase followed by a longer elimination phase *via* hepatic and renal routes. Moreover, they demonstrated that systemically injected exosomes can be delivered to tumor sites quickly. It was also reported that there was a difference in the biodistribution of exosomes according to the exosome-producing cells. Exosomes, which as both synthetic nanocarriers and cell-mediated drug delivery systems, enhanced tissue bioavailability and efficacy of relevant drugs (30–32). However, significant gaps remain in the complete understanding of the role of exosomes in diseases.

### EPC-Derived Exosomes

Previous studies have demonstrated affirmatively that EPCs are important therapeutic agents in the field of regenerative medicine, with potential utility in both cardiovascular therapies and other tissue engineering applications. Likewise, EPCs also synthesize and secrete functional exosomes that participate in angiogenesis and endothelium repair (33). EPCs-EXs were also found to display various beneficial therapeutic potentials compared to EPCs: regardless of political or ethical questions, they reduce the incidence of infectious diseases and tumor formation; they are available in large quantities, i.e., artificial exosomes can also be produced using clinical-grade synthetic lipids, recombinant proteins, and gene engineering in the future; and they do not induce significant immune responses after repeated transplantation. A comparison of EPCs and EPC-EXs is presented in Table 1. Exosomes from EPCs indeed carry a diversity of transcription factors, including exosome markers such as CD63, CD81, CD9, as well as many molecules that are signature characteristics of EPCs, such as CD34, VEGFR-2, and KDR (34). Exosomes from EPCs have also been found to contain lipids, proteins, mRNAs, precursor miRNAs (pre-miRNAs), miRNAs, and so on. Few studies have yet separated the functions of EPC-microvesicles (EPC-MVs) and EPC-EXs completely because of the limitations of isolation and identification methods. Wang et al. presented novel specific and sensitive methods for detecting EPC-MVs/EPC-EXs from cell culture medium and human plasma compared with previous techniques to differentiate exosomes by the combination of microbeads, fluorescence Q-dots, and nanoparticle tracking analysis (NTA) techniques. The average size of EPCs-MVs and EPC-EXs was 120 ± 1000 nm and 154 ± 59 nm by NTA analysis, which were in accordance with previous observations. EPC-specific antibody (CD34, KDR)-conjugated microbeads combined with fluorescence Q-dots were able to isolate and phenotype EPCs-MVs/EPC-EXs from biofluids (35, 36). EPC-EXs were released by abundant external stimuli, such as inflammatory conditioning of parental cells or hypoxia, indicating that the surrounding environment of EPCs would impact on exosome release. For example, EPCs decreased exosome release and downregulated the set of CD63, Alix, and Rab27a genes in response to diabetic-stimulated condition compared to normal condition (37). In another case, exosomes from endothelial colony-forming cells (ECFCs, a type of EPCs) in hypoxia significantly ameliorated cardiac fibroblasts by the reduction of miR-10b-5p, which targeted the fibrotic genes smad ubiquitin regulatory factor 1 (Smurf1) and histone deacetylase 4 (HDAC4), but did not exhibit this role in the normal condition (38). Moreover, moderate exercise could upregulate the levels of EPC-EXs and the amount of carried miR-126, and EPC-EXs hampered ECs apoptosis and angiogenic dysfunction through the modulation of miR-126/SPRED1/VEGF in a HG and hypoxia dual injury rat model; EPC-EXs thus improved the recovery of neurological function by alleviating acute brain cell apoptosis (39, 40). The previous observations suggested that healthy subjects and patients with different diseases released exosomes with different RNA and protein contents into the circulation, which could be measured as biomarkers. There were different levels of CD34^+^KDR^+^ EPC-EXs at different times during ischemic stroke, which may be used as biomarkers for diseases and indicators for the prognosis of and therapeutic efficacy for ischemic stroke (35).

**Table 1:** Comparison of EPCs and EPC-EXs in cell therapy.

	**EPCs**	**EPC-EXs**
Vasculogenesis	+	+
Angiogenesis	+	+
Anti-inflammatory	+	+
Anti-apoptosis	+	+
Cell regeneration	+	+
Anti-fibrosis	+	+
Disease biomarkers	+	+
Tumorigenicity	+	N
Tumor metastasis	+	N
“Capture” stenosis	+	N
Immunological rejection	+	N
Chromosomal variation	+	N
Emboli formation	+	N
Phenotype change	+	N

*+, data available; N, No data available*.

### miRNAs of EPC-Derived Exosomes

It has been clearly evidenced that miRNAs play a critical role in various pathological and physiological processes by regulating gene expression at the post-transcriptional level (41). Experimental studies identified that exosomes carry a distinctive repertoire of microRNAs (miRNAs) and other small non-coding RNAs, such as piRNAs, circRNAs, and lncRNAs (42). The proportion of miRNA in exosomes was found to be higher than that in their parent cells (43). There were various modes for cells to selectively sort miRNA into exosomes such as miRNA motif, sumoylated heterogeneous nuclear ribonucleoprotein (hnRNP)-dependent pathway, neural sphingomyelinase 2 (nSMase2)-dependent pathway, and miRNA induced silencing complex (miRISC)-related pathway (44–46). Exosomal miRNAs have been found to be more stable than free miRNAs and therefore had lasting effects on disease-related gene expressions. miRNAs from exosomes are considered important regulators of various of cellular processes involving cell–cell communication, such as cell survival and proliferation (47). EPC-EXs have abundant levels of miRNAs, which are a growing class of non-protein-coding single-strand RNAs consisting an average of only 22 nucleotides. miRNAs are derived from primary miRNAs, which are transcribed by RNA polymerase II from their own non-coding gene or from the introns of protein-coding genes. Primary miRNAs are cleaved into an average of 70-nucleotide-long miRNA precursor (pre-miRNA) by Drosa, which is then excised by Dicer into mature miRNAs that assemble with an argonaute protein to form the miRNA-induced silencing complex (RISC). The selective packaging of miRNAs in EPC-EXs (including EPC-MVs, since many earlier studies did not separate EPC-EXs from EPC-MVs) and their functions were also found important for disease treatment (Table 2) (38, 48–63). They are rich in cardioprotective and proangiogenic miRNAs, such as miR-126, miR-133, and miR-486. For instance, the miR-126 gene was indicated to be expressed in combination with its host gene Eg*f* l7, which plays a role in angiogenesis (64). Mounting evidence has revealed that miRNA-126 from EPC-EXs plays a critical role in cardiomyocyte protection, neovascularization, vascular homeostasis, repair, and thus the therapy of various vascular diseases. Sun et al. found that EPC-EXs loaded with miRNA-126 enhanced migration and angiogenesis in EPCs *in vitro* and significantly promoted thrombus resolution in an animal model of venous thrombosis (49). In a murine model of sepsis, miR-126-3p and miR-126-5p from EPC-EXs could maintain vascular homeostasis by reducing LPS-induced upregulation of vascular cell adhesion molecules-1 (VCAM-1) and high-mobility group box protein-1 (HMGB-1) in ECs, thereby reducing lung microvascular endothelial inflammation and dysfunction; the effects were reversed by transfecting with inhibitors of miR-126-3p and 5p (53). Other miRNAs, such as miRNA-18a, miR-21-5p, and miR-133, also participated in promoting endothelial dysfunction and inhibit myocardial fibrosis.

**Table 2:** Manuscripts demonstrating the functional effects of miRNA in EPCs-derived microvesicles/exosomes in cardiovascular diseases.

**Type**	**Stimulus**	**Biological effect**	**Recipient cell**	**Relevant molecular mechanism**	**Ref**.
miR-126	–	Promoted proliferation, angiogenesis, migration and acute pulmonary injury	Endothelial cells	Inhibited SPRED-1, activated the RAF/ERK signaling	(48)
	–	Promoted migration, tubulogenic activity, angiogenesis thrombosis resolution and recanalization	EPCs	Inhibited Pcdh7	(49)
	Lipopolysaccharide (LPS)	Reduced permeability, inflammation and pulmonary edema	Alveolar	Decreased HMGB-1, PIK3R2, and VEGFα, increased claudin1, claudin4 and occludin	(50)
	Lentivirus	Reduced apoptosis and promoted proliferation and migration	Osteoblast cells MC3T3-E1	Increased levels of Bcl-2 and p-Erk1/2 protein expression	(51)
	Uncontrolled diabetes healthy controls	Promoted migration, reduced apoptosis, and ROS production	EPCs	Increased VEGFR2	(52)
miR-126-5p, miR-126-3p	Sepsis	Prevented microvascular dysfunction, improved sepsis outcomes	Microvascular	Decreased HMGB-1 and VCAM1	(53)
	–	Improved proliferation, migration, and angiogenic capacity	Endothelial cells	Increased VEGFA, bFGF, TGFB1, and ANG	(54)
miR-133	Hypoxia/reoxygenation	Promoted angiogenesis and inhibited MEndoT of cardiac fibroblasts	Cardiac fibroblasts	Increased CD31, VE-cadherin and vWF, decreased α-SMA, N-cadherin, vimentin, and collagen I, increased YBX-1, SYNCRIP and hnRNPA2B1	(55)
miR-21-5p	Balloon injury	PROMOTED proliferation, migration, angiogenic capacity and EC repair.	Endothelial cells	Suppressed THBS1	(56)
miR-18a	Hypoxia/reoxygenation	Decreased apoptosis, promoted angiogenesis	Aging endothelial cells	Decreased Nox2, increased nitric oxide and eNOS, activated the miR-18a/eNOS/NO pathway	(57)
miR-486-5P	Hypoxia	Blocked apoptosis, reduced ischemic kidney injury	Endothelial cells	Decreased PTEN	(58)
miR-221-3p		Promoted proliferation, angiogenesis, and skin wound healing in diabetic mice	Diabetic skin wounds	Decreased p27, caspase-3, E-selectin, c-Jun N-terminal kinase involved in the AGE-RAGE signaling pathway.	(59)
miR-124	–	Reversed the migration and osteoclastic differentiation, enhanced fracture healing	Bone marrow-derived macrophages	Increased lncRNA-MALAT1 and ITGB1	(60)
miR-10b-5p	Normoxia	Anti-fibrotic and reduced cardiac fibrosis	Cardiac fibroblasts	Decreased HDAC4 and Smurf1	(38)
miR-210	Hypoxia/reoxygenation(H/R)	Reduced H/R-induced endothelial cell apoptosis, ROS overproduction and angiogenic dysfunction, improved mitochondrial function	Endothelial cells	Decreased mitochondrial fragmentation, elevated MMP and ATP level	(61)
	Oxygen-glucose deprivation (OGD)	Promoted proliferation	EPCs	Increased Ca2+ fluctuation	(62)
miR-137	Oxyhemoglobin	Reduced the number of apoptotic neurons	SH-SY5Y cells	Increased COX2 and PGE2	(63)

## Therapeutic Potential of EPC-Derived Exosomes for The Cardiovascular System

### Anti-inflammation and Anti-oxidation

CVDs are closely associated with inflammation, oxidative stress, and redox signaling. Persistent low-grade inflammation was shown to cause immunosenescence within the aging process, which contributed to endothelial dysfunction, atherosclerosis, activating RAAS, cardiac remodeling, and cardiovascular complications (65). Oxidative stress leads to eNOS uncoupling, whose functional manifestation is endothelial dysfunction (66). Thus, it is worth noting that targeted anti-inflammation and anti-oxidation therapy can lower cardiovascular mortality. It is well-established that exosomes of various cellular origin, including EPCs, participate in the inhibition of inflammation response to repair tissue in *in vitro* and *in vivo* models (67, 68). Zhou et al. demonstrated that EPC-EXs treatment significantly attenuated these increases of inflammatory mediators such as tumor necrosis factor (TNF)-α, interleukin (IL)-6, IL-1β, interferon (IFN), macrophage inflammatory proteins (MIP)-1, MIP-2, monokine induced by gamma interferon (MIG), and interferon gamma-induced protein (IP)-10 (50, 53). One study indicated that ECFC-derived exosomes (ECFC-EXs) were enriched in exosome markers tumor susceptibility gene101 (TSG101) and CD63. Treatment with ECFC-EXs blocked hypoxia/reoxygenation (H/R)-induced increases due to the expression of the proinflammatory protein intercellular cell adhesion molecules-1 (ICAM-1) and endothelial cell apoptosis (69). Interleukin (IL)-10 is an anti-inflammatory cytokine that suppresses macrophage and proinflammatory Th17 T-cell responses by inhibiting the inflammatory cytokines IL-6, IL-12, and IL-23. Another study also demonstrated that EPCs augmented the LPS-induced production of macrophage IL-10 and expression of miRNA-126 and miRNA-125b, which regulated EC function and inflammation, thus promoting the decrease of lung vascular leakage, liver, and kidney injury in sepsis *in vivo* (70). According to various studies, EPC-EXs provided anti-oxidative properties through reducing reactive oxygen species (ROS) production and enhancing eNOS expression (52, 57). The investigations revealed that EPC-EXs exerted protective effects through the inhibition of inflammation reaction and the promotion of anti-oxidation.

### Anti-apoptosis

The apoptosis (programmed cell death) of cells has been previously identified as an important process in a variety of CVDs, including atherosclerosis, heart failure, ventricular remodeling, pulmonary arterial hypertension, and other peripheral arterial diseases (71–73). In hypoxic circumstances, the cell initiates a cascade of events such as energy deprivation, radical formation, and in particular ROS generation that lead to apoptotic cell death (74). Many emerging studies have suggested that EPC-EXs presented cell protective features of anti-apoptosis by modulating miRNAs and a variety of downstream signaling pathways (57, 61). However, the detailed underlying mechanisms of the anti-apoptotic effect of EPC-EXs remain unclear.

### Inhibition of Endothelial-to-Mesenchymal Transition (EndMT) and Cardiac Fibrosis

Excessive cardiac fibrosis is a significant problem in nearly all types of CVDs. Cardiac fibrosis originates from fibroblast proliferation and strong activation, and EndMT partially enhances the process of fibrosis in organs including in the heart. EndMT is a process where ECs reduce the expression of endothelial genes/proteins (CD31, VE-Cadherin) and increase the expression of mesenchymal genes/proteins such as alpha-smooth muscle actin (α-SMA), vimentin, Pro-collagen, and fibroblast-specific protein-1 (FSP-1). The TGF-β signaling system activates SMAD proteins from complexes and interacts with key regulators of EndMT: SNAI1, SNAI2, ZEB1, ZEB2, KLF4, TCF3, and TWIST. These interactions culminate in chromatin rearrangements and transcription factor binding to endothelial, mesenchymal, and other relevant gene promoter regions that induce EndMT (5, 75). Signaling molecules involving Wnt/β-catenin, endothelial FGFR1 signaling, mitochondrial protein endothelial SIRT3, and nuclear receptor endothelial glucocorticoid receptor are also the endogenous anti-EndMT molecules and their loss leads to activation of EndMT events in organs. eNOS uncoupling and inflammatory cytokines reduce endothelial FGFR1 and endothelial glucocorticoid receptor expression and activate TGF-β and Wnt signaling. A previous study has demonstrated that loss of SIRT3 in ECs disrupted the EC homeostasis, displayed a higher level of TGFβ-smad3 signaling, and displayed defective metabolism-associated EndMT (76). N-acetyl-seryl-aspartyl-lysyl-proline (AcSDKP) is an endogenous anti-fibrotic peptide, which is associated with fibroblast growth factor receptor1 (FGFR1). Li et al. have investigated that endothelial FGFR1 deficiency in diabetic mice resulted in severe organ fibrosis in both the kidney and heart *via* the induction of AcSDKP-resistant EndMT (77). Mesenchymal-to-endothelial transition (MEndoT) could make fibroblasts obtain the functions of ECs and make them participate in angiogenesis in the cardiac injury area, which could reverse cardiac fibrosis (78). Recent evidence suggested that EPC-EXs promote fibroblast angiogenesis and MEndoT through the intercellular transfer of miR-133, thereby attenuating cardiac fibrosis (41). Another investigation confirmed that EPC-EXs enhanced the proliferation and angiogenesis of cardiac fibroblasts *in vitro* and increased the expression of the EC-specific markers, including CD31 and VEGF-2, and decreased the expression of proteins involved in fibrosis, such as α-SMA, vimentin, collagen I, TGF-β, TNF-α, and HMGB1. Therefore, EPC-EXs promoted the proliferation and angiogenesis of cardiac fibroblasts by inhibiting EndMT and decreasing the expression of HMGB1 (79). EPC-EXs inhibit the progression of cardiac fibrosis by mediating homeostasis of EndMT and MEndoT.

### Cell Regeneration, Anti-vascular Remodeling, and Angiogenesis

Vascular remodeling, which is a typical pathological characteristic of various CVDs, such as atherosclerosis, hypertension, pulmonary hypertension, and myocardial hypertrophy (80–82), is a critical target in the treatment of CVDs. Therapeutic angiogenesis offers another promise to improve blood supply in ischemic CVDs (83, 84). It was indicated that human EPC-EXs enhanced the proliferation and migration of endothelial cells *in vitro* and promoted vascular repair in rat models of balloon injury by upregulating ECs function *in vivo* (85). In another rat model of balloon-induced carotid artery injury, it was demonstrated that the administration of EPC-EXs potentiated re-endothelialization after endothelial damage probably through inhibiting thrombospondin-1 (THBS1) and delivering miR-21-5p (56). Chen et al. demonstrated that EPC-EVs (including EPC-MVs and EPC-EXs) enhanced peri-infarct angiogenesis and hemodynamics after MI (86). All of these scholars paid attention to the importance of anti-vascular remodeling and angiogenesis. Meanwhile, more effort should be made to study the molecular mechanisms of anti-vascular remodeling and angiogenesis by EPCs-EXs.

### Function of Gene-Modified EPC-EXs

With extensive ongoing research on EPC-EXs, many researchers have focused on the superiority of EPC-EXs combined with gene transfer for therapeutic angiogenesis and vasculogenesis. Many studies targeted on consequences of ACE2 transduced EPC-EXs on ECs *in vitro* and neovascularization *in vivo*. The studies found that ACE2 gene transfer of EPC-EXs decreased the apoptosis of injured ECs, ROS production, mitochondrion fragmentation, Nox2 and Nox4 expression, and increased ECs function, MMP, and ATP levels through downregulating Nox2 and upregulating eNOS *in vitro* (57, 87). Furthermore, Wang et al. further explored the effect of combining EPC-EXs with ACE2 gene transfer in a C57BL/6 mice model of intracerebral hemorrhagic stroke (ICH) *in vivo*. They found a statistically significant decrease of hemorrhage volume for the ACE2-EPC-EXs rather than the EPC-EXs group. ACE-EPC-EXs also improved neurological deficit and BBB permeability, alleviated brain edema, downregulated the expressions of TNF-a and NFrB and upregulated the IrBa level (88). miR-126 has been a further target agent for gene modification to enhance the vasculogenic properties of EPC-EXs (49, 51, 89). For instance, transfer of miR-126 by EPC-EXs reduced apoptosis and promoted the proliferation and migration of MC3T3-E1 cells *in vitro* (51). Transfer miR-126 of EPC-EXs decreased infarct volume, increased cerebral blood flow (CBF) and cerebral microvascular density (MVD), promoted angiogenesis and neurogenesis, and downregulated cleaved caspase-3 and VEGF2 more significantly than EPC-EXs in a db/db type II diabetic mice model of middle cerebral artery occlusion (MCAO) surgery for inducing ischemic stroke *in vivo* (89). Sun et al. demonstrated that EPC-EXs loaded with miR-26 promoted thrombus and recanalization by elevating Pcdh7 mRNA expression (49). To this end, the transfer of single genes and multiple genes to enhance EPC-EXs function, as well as the understanding of the underlying molecular and cellular mechanisms should be researched more extensively.

## Therapeutic Effects of EPC-Derived Exosomes in CVDs

EPC-EXs play an essential role in EPCs-based therapies of CVDs, including atherosclerosis, MI, and reperfusion injury. EPCs-EXs were shown to exhibit a therapeutic effect similar to EPCs transplantation, and some investigations also demonstrated their effects of promoting the prognosis of CVDs in animal models *in vivo* (49, 90–92) (Table 3).

**Table 3:** Therapy by exosomes/microvesicles for cardiovascular repair in animal *in vivo* models.

**Species/type of injury or *in vivo* assay**	**Dosage**	**Therapeutic type**	**Function, highlights**	**Ref**.
C57/BL6 male mice/deep venous thrombosis (inferior vena cava)	300 μg of Exo or miR-126-Exo	Were transplanted into the femoral vein *in situ*.	Promoted the migration and angiogenesis of EPCs, improved thrombus organization and recanalization	(49)
Mice/left anterior descending coronary artery (LAD)	2 × 10^9^ particles(exosomes)	Were transplanted intramyocardially into the left ventricular wall (border zone) at three different locations immediately after left anterior descending ligation	Decreased MI scar size and promoted neovascularization	(90)
Female SD rats/balloon injury (left common carotid artery)	2 × 10^12^ exosome particles	No data available	Accelerated re-endothelialization of the injured arteries	(91)
Male SD rats/balloon injury (left common carotid artery)	30 μg exosomes	Intravenously injected	Promoted EC repair; inhibited neo-intimal hyperplasia	(92)

### Atherosclerosis

Atherosclerosis is a serious vascular disease characterized by endothelial dysfunction, inflammation, and the formation of plaques. The latter contain lipids, extracellular matrix, mesenchymal cells, and immune cells. The process of EndMT makes ECs acquire the markers and functions of mesenchymal cells and thus can act as a source of mesenchymal cells in atherosclerotic plaques (93). Several prior studies reported that EPC-EXs had the crucial functions of promoting ECs dysfunction, reducing oxidative stress, elevating eNOS expression (57), and inhibiting EndMT (78). In a mouse model of atherosclerosis treated with EPC-EXs, the atherosclerotic plaques abundantly decreased. Anti-atherosclerosis processes might include the regulation of miRNAs expression of EPC-EXs; decrease of the levels of oxidative stress factors malondialdehyde (MDA) and superoxide dismutase (SOD) and the inflammatory factors ICAM-1, IL-8, and C-reactive protein (CRP); and the change of high K^+^ solution- and Phe-induced vasoconstriction and endothelium-dependent vasodilation in the thoracic aorta (94).

### Myocardial Infarction (MI)

MI, a detrimental consequence of acute coronary occlusion, is featured by inflammation, the apoptosis of cardiomyocytes and oxidative stress, which induce vasodilatation and increase neovascularization. In the past decades, various therapeutic strategies have been tested to find a more effective treatment for CVDs. Notably, cell therapy has gradually become an attractive and effective treatment method for CVDs (95–97). With the emergence of studies on EPC-EXs, exosome secretion by various cell types, including cardiomyocytes (CMs), ECs, fibroblasts, and circulating progenitor cells (CPCs), have been demonstrated to provide protective prognosis in MI. In a mouse MI model, EPC-EXs were injected intramyocardially into the left ventricular wall (border zone) at three different locations immediately after left anterior descending ligation. In the IL-10 knockdown group, EPC-EXs were enriched in inflammation-related proteins featuring a two- to fourfold increase in Integrin Linked Kinase (ILK) expression, and were shown to activate the NF-κB pathway in recipient cells and enhance inflammatory response by upregulating inflammatory genes, while wild-type EPC-EXs showed the opposite effects. Wild-type EPC-EXs also improved the left ventricular cardiac function, significantly reduced cardiomyocyte apoptosis, decreased MI scar size and promoted neovascularization compared with IL-10 knockdown EPC-EXs (90). In another rat model of MI, it was revealed that EPC-EVs (including EPC-MVs and EPC-EXs) (injections around the border zone of the infarcted area) delivered into the ischemic myocardium *via* an injectable hydrogel enhanced peri-infarct angiogenesis and myocardial hemodynamics, and the therapeutic efficiency and efficacy of myocardial preservation was greatly increased by a shear-thinning gel (86).

### Reperfusion Injury

Ischemia–reperfusion injury (I/RI) might result from increasing mortality and morbidity by irreversible structural damage and organ dysfunction in a large number of diseases, such as MI, stroke, and transplantation (98). The production of free radicals in I/RI is attributed to myocardial injury, which has three forms: myocardial stunning, reperfusion arrhythmia, and myocardial necrosis. Many investigations have deepened our insight into the mechanisms and therapeutic strategies for myocardium I/IR, including the role of exosomes (99, 100). EPC-EXs were found to reduce apoptosis, ROS overproduction, and angiogenic dysfunction; decrease mitochondrial fragmentation; elevate MMP and ATP level; and improve mitochondrial mfn2 and drp1 dysregulation in endothelial cells (61). ACE2-EPC-EXs exhibited greater anti-oxidative and anti-apoptotic effects on aging ECs than on young ECs subject to H/R injury through carrying miR-18a and subsequently downregulating the Nox2/ROS pathway (57). Further experiments should also be conducted to examine the protective roles and mechanisms of EPC-EXs in I/RI *in vivo*.

### Perspectives and Future Direction

Rapid detection of CVDs is the cornerstone of improving prognosis and preventing further comorbidities and complications. The possibility to isolate and characterize EPC-EXs from bodily fluids makes them very attractive diagnostic markers. Many exosome-based cancer diagnostic kits have been developed quickly in recent years. However, in the field of cardiovascular medicine, EPC-EXs as diagnostic markers are still an unexplored world that we are committed to pioneer. In addition, it is more attractive to use EPC-EXs as a therapeutic drug rather than conventional EPC transplantation. However, the same composition of exosomes expresses various pathophysiological functions under different microenvironments *in vivo*; how to preserve the biological activity of cytokines, proteins, and miRNAs in exosomes and deliver them to target sites is a big challenge for us now. The biodistribution, as well as the long-term effects and safety of administered EPC-EXs, would need to be explored and controlled.

As mentioned above, the promise and excitement surrounding EPC-EXs in CVDs can be manifested daily by previously reported studies. Although the field of EPC-EXs has much to be developed, the exploration and specific application of EPC-EXs and potential treatment will continue to be a rapidly advancing focus for cardiovascular researchers. Exosome-based approaches could “take EPCs out of cell therapy.”

## Author Contributions

All authors listed have made a substantial, direct and intellectual contribution to the work, and approved it for publication.

## Conflict of Interest

The authors declare that the research was conducted in the absence of any commercial or financial relationships that could be construed as a potential conflict of interest.

## Publisher's Note

All claims expressed in this article are solely those of the authors and do not necessarily represent those of their affiliated organizations, or those of the publisher, the editors and the reviewers. Any product that may be evaluated in this article, or claim that may be made by its manufacturer, is not guaranteed or endorsed by the publisher.
